# Recurrence of IgA nephropathy after kidney transplantation: experience from the Swiss transplant cohort study

**DOI:** 10.1186/s12882-022-02802-x

**Published:** 2022-05-10

**Authors:** Cédric Jäger, Susanne Stampf, Karen Molyneux, Jonathan Barratt, Déla Golshayan, Karine Hadaya, Uyen Huynh-Do, Francoise-Isabelle Binet, Thomas F Mueller, Michael Koller, Min Jeong Kim

**Affiliations:** 1 Clinic for Nephrology and Dialysis, Cantonal Hospital Basel-Land, Liestal, Switzerland; 2 Clinic for Transplantation immunology and nephrology, University Hospital Basel, Basel & Swiss Transplant Cohort Study (STCS), Basel, Switzerland; 3 Department of Cardiovascular Sciences, University of Leicester, Leicester, UK; 4 Transplantation Center, CHUV University Hospital, Lausanne, Switzerland; 5 Division of Nephrology, Geneva University Hospital, Geneva, Switzerland; 6 Department of Nephrology and Hypertension, University of Bern, Bern, Switzerland; 7 Division of Nephrology/Transplantation Medicine, Kantonsspital St. Gallen, St. Gallen, Switzerland; 8 Division of Nephrology, University Hospital Zürich, Zürich, Switzerland; 9 Division of Nephrology, Cantonal Hospital Aarau, Aarau, Switzerland

**Keywords:** IgA nephropathy, Kidney transplantation, Recurrent glomerulonephritis, Transplant outcome, Predictive markers

## Abstract

**Background:**

Recurrence of IgA nephropathy (IgAN) after kidney transplantation occurs in about 30% of patients. The relevance of recurrence for the long-term graft survival is expected to increase, since graft survival continues to improve.

**Methods:**

In a nested study within the Swiss Transplant Cohort Study the incidence of IgAN recurrence, predictive factors, graft function and graft and patient survival were evaluated. Serum concentration of total IgA, total IgG, Gd-IgA1 and IgA-IgG immune complex were measured using ELISA-based immunologic assays.

**Results:**

Between May 2008 and December 2016, 28 women and 133 men received their kidney allograft for end-stage kidney disease due to IgAN in Switzerland. Over a median follow-up time of 7 years after transplantation, 43 out of 161 patients (26.7%) developed an IgAN recurrence, of which six (13.9%) had an allograft failure afterwards and further four patients (9.3%) died. During the same follow-up period, 6 out of 118 patients (5%) each experienced allograft failure or died without prior IgAN recurrence. After 11 years the risk for IgAN recurrence was 27.7% (95%-CI: 20.6–35.3%). Renal function was similar in patients with and without recurrence up to 7 years after transplantation, but worsened thereafter in patients with recurrence (eGFR median (interquartile range) at 8 years: 49 ml/min/1.73m^2^ (29–68) vs. 60 ml/min/1.73m^2^ (38–78)). Serum concentration of total IgA, total IgG, Gd-IgA1 and IgA-IgG immune complex within the first year posttransplant showed no significant effect on the recurrence of IgAN. Younger recipients and women had a higher risk of recurrence, but the latter only in the short term.

**Conclusions:**

Our study showed a recurrence risk of 28% at 11 years after transplantation, which is consistent with previous literature. However, the predictive value of known biomarkers, such as serum Gd-IgA1 and IgA-IgG IC, for IgAN recurrence could not be confirmed.

**Supplementary Information:**

The online version contains supplementary material available at 10.1186/s12882-022-02802-x.

## Background

On the natural course of IgA nephropathy (IgAN), 20–40% of patients develop end-stage kidney disease (ESKD) within 25 years. Kidney transplantation is one of the therapeutic options offered to these patients; however, recurrence of IgAN on the graft can occur with a frequency ranging from 20 to 60% and the graft survival is significantly worse in patients with recurrent IgAN [1–3]. As graft survival continues to improve, the relevance of IgAN recurrence for the long-term graft survival is expected to increase in the future [4]. Several factors, such as younger age of recipients, a rapid progression of the original disease, uses of anti-thymocyte globulins and certain HLA-types have been reported to be predictive for recurrence [1, 5].

In the case of recurrence, there is currently no specific treatment option. However, beneficial effect of angiotensin-converting enzyme inhibitor has been reported [6] and a longer exposure to steroids was associated with a lower rate of IgAN recurrence [7]. Better identification of patients with increased risk of recurrence and treatment optimization is therefore desired to improve the graft survival in kidney transplant recipients with IgAN as the etiology of ESRD.

Patients with IgAN have increased serum levels of IgA1 with truncated galactose-deficient hinge region *O*-glycans (Gd-IgA1) and IgA1 eluted from the glomeruli in renal tissue of patients with IgAN has the same glycosylation aberrancy [8]. Several studies have shown that the level of serum galactose-deficient IgA1 (Gd-IgA1) at the time of renal biopsy significantly correlates with disease progression [9, 10]. The level of Gd-IgA1 was also associated with more severe histologic findings [11]. Not only the level of Gd-IgA1, but also the serum levels of IgG and IgA autoantibodies seem to associate with the progression of IgAN [8].

In a single centre study with 60 kidney transplant recipients with IgAN, pre-transplantation serum Gd-IgA1 and IgA-IgG immune complexes were highly predictive for IgAN recurrence [12]. In another previous study, however, there was no significant association of serum Gd-IgA1 level with the recurrence of IgAN in 61 patients [13]. Another most recent study reported elevated serum levels of anti-Gd-IgA1-specific IgG autoantibody at transplant to be an independent risk factor, whereas serum Gd-IgA1 did not show any association with IgAN recurrence [14].

In this study, we report IgA nephropathy recurrence rates, predictive values of known biomarkers for recurrence, and transplant outcomes in patients with IgAN in Swiss transplantation cohort.

## Methods

### Study design and data source

The current study was nested within the Swiss Transplant Cohort Study (STCS). The STCS is a prospective, nationwide cohort enrolling all solid organ transplant (SOT) recipients at six transplant centres in Switzerland (Basel, Bern, Geneva, Lausanne, St. Gallen, and Zurich) since May 2008. In the STCS, clinical and laboratory data are prospectively collected via electronic case report forms using standard definitions. After STCS enrolment, regular cohort visits are scheduled at six- and twelve-month post-transplant, and yearly thereafter. Blood and urine samples are collected alongside each transplantation right before transplantation, six- and twelve-month post transplantation. Patients provided written informed consent to the STCS and the ethics committee affiliated with each transplantation centre approved the STCS and the current nested study.

### Study participants

Of 2464 renal transplant recipients enrolled between May 2008 and December 2016 in the STCS, 161 had a biopsy-proven IgAN as underlying disease leading to renal transplantation and were included in our study population. Further inclusion criteria were defined as age at transplantation older than 18 years and given written informed consent to STCS participation. Instances of renal transplantation for IgAN with primary non-function were excluded. The follow-up period ended with graft failure or death, whichever occurred first, or latest at the censoring date end of 2019.

### Outcomes and diagnosis of recurrence

Primary outcome was a recurrence of IgAN after transplantation. The diagnosis of recurrence was made centrally by “adjudicated final diagnosis” procedure. In this procedure, two independent nephrologists reviewed all available medical records - patient history, results of laboratory testing including blood and urine analysis, and biopsy results pertaining to the patient from the time of transplantation to the time of the analysis. The two nephrologists were blinded to the investigated biomarkers. In situations of disagreement about the diagnosis, cases were reviewed and adjudicated in conjunction with a third nephrologist. Three out of six transplant centres performed surveillance biopsy according to the local biopsy policy and both surveillance and diagnostic biopsies were included in the analysis. Biopsy assessment was performed in each centre by the local pathologists. Recurrence of IgAN was defined as mesangial IgA deposits in kidney biopsy, with or without mesangial expansion and/or endocapillary hypercellularity. Cases of recurrent glomerular hematuria and / or proteinuria and not otherwise explained impaired graft function were defined as clinically suspected recurrence and also included in the analysis. Patients without recurrence are defined as the absence of histological recurrence on the surveillance and diagnostic biopsies and the absence of glomerular hematuria and proteinuria at the last follow-up.

Secondary outcomes were identification of risk factors for IgAN recurrence, and post-transplant renal function (eGFR calculated by CKD-EPI equation), proteinuria and graft- and patient survival in patients with and without IgAN recurrence.

### Measurements

An ELISA-based immunologic assay was used to measure concentration levels of four biomarkers in our patients’ serum samples: total IgA, total IgG, galactose-deficient IgA1 (Gd-IgA1) and IgA-IgG immune complexes (IgA-IgG IC). All measurements were performed centrally at the University of Leicester. The measurements methods were described previously in detail elsewhere [15, 16].

Briefly, total IgA was quantified by specific ELISA using polyclonal rabbit anti-human α-heavy chains (DAKO) as primary antibody, NIBSC serum standard (cat. No. 67/099), HRP-conjugated polyclonal anti-IgA as secondary antibody, and OPD substrates (Thermo Fisher). Total IgG was quantified by specific ELISA using rabbit anti-human IgG (DAKO A0423) as primary antibody, NIBSC serum standard (cat. No. 67/099), HRP-conjugated polyclonal anti-IgG as secondary antibody, and OPD substrates. The results of IgA and IgG concentration was given as mg/ml.

Levels of Gd-IgA1 were measured by lectin-binding assay using rabbit anti-human IgA (DAKO A0262), neuraminidase enzyme (New England Bio Labs P0720L), GalNAc specific biotinylated *Helix pomatia* agglutinin (Sigma, L6512), streptavidin-HRP (R&D DY998) and OPD substrates. The results were expressed as optical density (OD).

Serum IgA-IgG complexes were quantified by specific ELISA using AffiniPure F (ab’)_2_ Fragment Goat anti-human serum IgA, α-chain specific (Jackson-Immuno Research) as the capture antibody, HRP-conjugated Polyclonal Rabbit Anti-human IgG (DAKO) as the secondary antibody and OPD substrate. The results were expressed as optical density (OD).

Serum creatinine and proteinuria (protein-creatinine-ratio in spot urine sample, mg/mmol) were collected from routine lab read-outs from the hospitals at each STCS follow-up visit and at the time of recurrence.

### Statistical analysis

Clinical and demographic characteristics and donor-related information in the study population were descriptively shown. The distribution and median concentration levels of the four biomarkers was illustrated at time of transplantation, six- and twelve-month post-transplant. The cumulative incidence function method was used to estimate the probability of IgAN recurrence treating graft failure and patients’ death as competing events. The effect of the biomarkers on the post-transplant IgAN recurrence was assessed in separate event-specific Cox proportional hazard (PH) models. We considered baseline biomarker data and, depending on the data, time-updated measurements. In addition, a sensitivity analysis including biopsy-proven IgAN recurrence only was performed. Values of protein−/creatinine-ratio and estimated GFR over time were additionally illustrated using box plots.

All statistical analyses were performed using the statistical software R version 4.0.4.

## Results

### Patient and donor baseline characteristics

Between May 2008 and December 2016, 28 (17.4%) women and 133 men received their kidney allograft for ESKD due to biopsy-proven native kidney IgAN in Switzerland. Almost all patients were Caucasian (91.9%). Their median age was 48 years and that of donors 53. There were more female (53%) and living donors (59%), and 65% of living donations were from relatives. Twenty-two patients (13.7%) received their kidney allograft for the second or third time. At the time of transplantation, 18 patients (11.2%) were treated with immunosuppressants (15 patients from their previous transplantation, 2 according to the ABO incompatible transplantation protocol, and 1 for her rheumatoid arthritis) (Table 1).

**Table 1:** Baseline characteristics of renal transplant recipients with IgA nephropathy as underlying disease leading to TX and their donors in the study population

Recipient and donor baseline characteristics	All patients (***n*** = 161)	R group (***n*** = 43)	NR group (***n*** = 118)
Median age at TX (years) [IQR]	48 [38, 60]	39 [30, 53]	50 [42, 61]
Female, N (%)	28 (17.4%)	10 (23.3%)	18 (15.3%)
Caucasian, N (%)	148 (91.9%)	37 (86%)	111 (94.1%)
History of prior kidney TX, N (%)	22 (13.7%)	7 (16.3%)	15 (12.7%)
KRT prior to TX, N (%)			
- hemodialysis	95 (59%)	25 (58.1%)	70 (59.3%)
- peritoneal dialysis	28 (17.4%)	7 (16.3%)	21 (17.8%)
- pre-emptive TX	38 (23.6%)	11 (25.6%)	27 (22.9%)
Immunosuppression immediate before TX, N (%)	18 (11.2%)	5 (11.6%)	13 (11%)
Median age at donation [IQR]	53 [42, 62]	49 [42, 58]	55 [42, 63]
Female donors, N (%)	85 (52.8%)	25 (58.1%)	60 (50.8%)
Living donation, N (%)	95 (59%)	26 (60.5%)	69 (58.5%)
- from relatives	62 (65.3%)	17 (65.4%)	45 (65.2%)
**Peri-transplant information**			
Early allograft dysfunction, N (%)	15 (9.3%)	2 (4.7%)	13 (11%)
Median cold ischemia time (hours) [IQR]	2.1 [1.2, 9.8]	1.5 [1.1, 10.7]	2.2 [1.3, 9.1]
Induction therapy with ATG/Thymoglobulin, N (%)	26 (16.1%)	8 (18.6%)	18 (15.3%)
Immunosuppressive regimen, N (%)			
- TAC-based	135 (83.9%)	41 (95.3%)	94 (79.7%)
- Others	26 (16.1%)	2 (4.7%)	24 (20.3%)
Presence of HLA antibodies, N (%)	74 (46%)	23 (53.5%)	51 (43.2%)
Presence of DSA, N (%)	20 (12.4%)	7 (16.3%)	13 (11%)
- Class I	5 (25%)	1 (14.3%)	4 (30.8%)
- Class II	11 (55%)	2 (28.6%)	9 (69.2%)
- Class I + II	4 (20%)	4 (57.1%)	0 (0%)
HLA full match, N (%)	13 (8.1%)	3 (7%)	10 (8.5%)
Mean number of HLA A/B/DR mismatch [min, max]	3.8 [1, 6]	3.7 [1, 6]	3.8 [1, 6]
CMV high-risk constellation (D+/R-), N (%)	19 (11.8%)	6 (14%)	13 (11%)
Administration of RAAS blockade (ACE inhibitor, ARB), N (%)	55 (34.2%)	17 (39.5%)	38 (32.2%)

*Abbreviations*:*TX* transplantation, *IQR* interquartile range, *R/NR* recurrence/non-recurrence, *KRT* kidney replacement therapy, *IQR* interquartile range, *ATG* anti-thymocyte globulin, *TAC* tacrolimus based therapy as combination of prednisone, tacrolimus and mycophenolate mofetil (MMF), *HLA* human leukocyte antigen, *DSA* donor specific antibodies, *CMV* cytomegalovirus, *D+* seropositive donor, *R*- seronegative recipient, *RAAS* renin-angiotensin-aldosterone system, *ACE* angiotensin-converting-enzyme, *ARB* angiotensin receptor blockers

The proportion of early allograft dysfunction was low (9%) and the median cold ischemia time was 2 hours. Most patients received tacrolimus-based immunosuppressive regimen (83.9%). Donor-specific antibodies (DSA) were present at the time of transplantation in 20 patients (12.4%), and 26 patients (16.1%) received induction therapy with anti-thymocyte globulin or thymoglobulin. Mean number of HLA mismatches in A, B, and DR loci was 3.8.

One-third of all (55/161) patients were under RAAS blockade at the time of transplantation.

### Recurrence of IgA nephropathy and transplantation outcomes

**Fig. 1:**
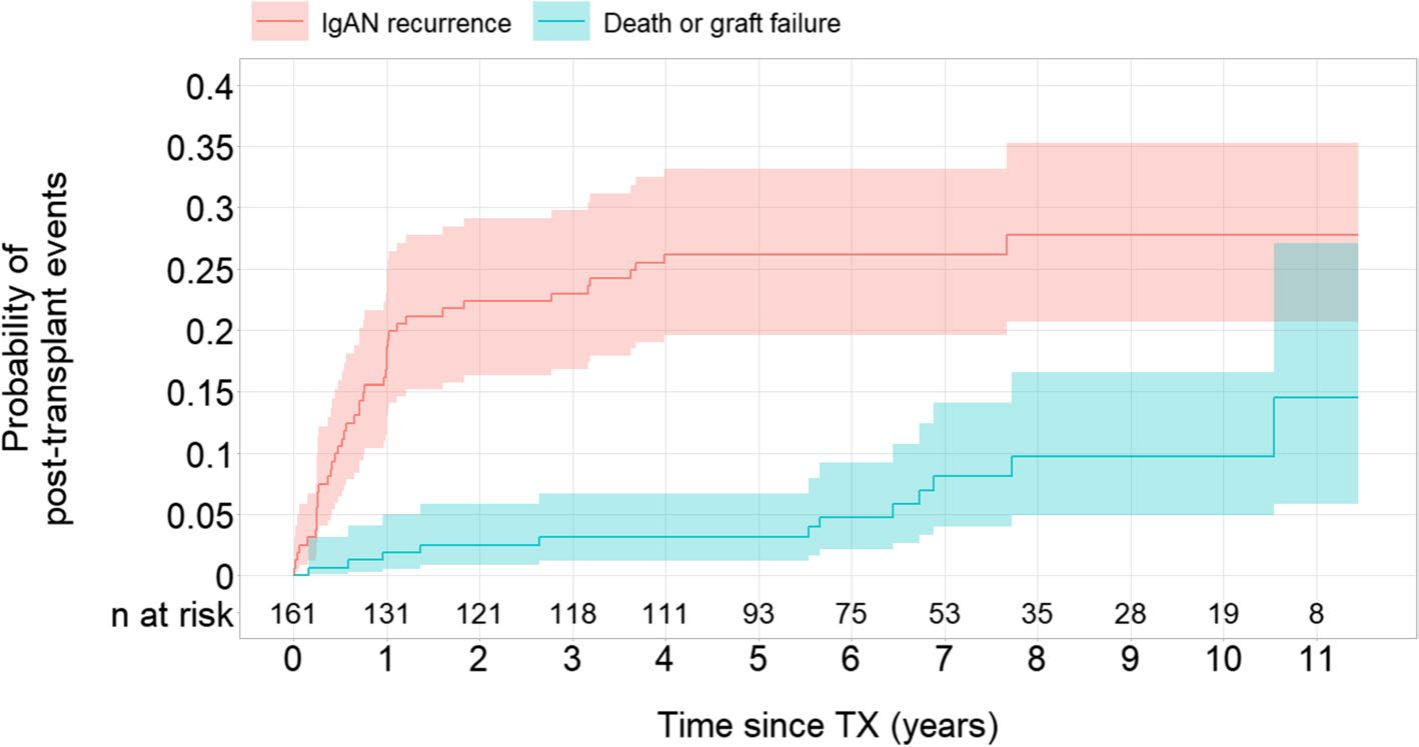
Probability of IgAN recurrence and death or graft failure post-transplant in the study population. Abbreviations: TX – transplantation; IgAN – IgA nephropathy

Over a median follow-up period of 7.1 (interquartile range, 5.7–9.2) years, 43 patients (26.7%) developed recurrence of IgAN. The risk for IgAN recurrence was 27.7% (95%-CI: 20.6–35.3%) after 11 years (Fig. 1). Median time to recurrence was 8.5 months (interquartile range, 3.2–12.8 months). Twenty-four events were diagnosed by surveillance biopsy (56%), 13 by diagnostic-biopsy (30%) and 6 were clinically suspected without performing a biopsy (14%).

Six patients (13.9%) with recurrence had subsequent allograft failure: 4 patients due to the recurrence and 2 patients due to an immunological reason. Four patients (9.3%) died after recurrence: 2 patients due to cardiovascular disease, and one patient each due to respiratory infection and malignancy. Median time to graft failure or death post-recurrence was 4.8 years (interquartile range, 1.4–6.5 years). During the same follow-up period, 6 out of 118 patients without IgAN recurrence (5%) developed allograft failure: 4 patients due to an immunological reason, and one patient each due to a transplant kidney pyelonephritis and an unknown etiology. Six patients without recurrence (5%) died: 3 patients due to cardiovascular disease, 2 patients due to a malignancy and 1 patient due to an accident (Table 2). The probability of recurrence free allograft failure and death in the study population was low at the end of follow-up (total 14.5%; allograft failure 5.4% and death 9.1%) (Fig. 1, Supplementary data Fig. S1).

**Table 2:** Description of primary and competing outcomes in the study population of 161 renal transplant recipients with IgA nephropathy as underlying disease leading to TX

**Follow-up information**	
Median follow-up time (years) [IQR]	7.1 [5.7, 9.2]
**Primary outcome**	
Recurrence of IgA nephropathy, N (%)	43 (26.7%)
- median time from TX to IgAN recurrence (months) [IQR]	8.5 [3.2, 12.8]
- graft failure / death *post* IgAN recurrence, N (%)	6 (14%) / 4 (9.3%)
Median time to graft failure or death post IgAN recurrence (years) [IQR]	4.8 [1.4, 6.5]
**Competing (recurrence-free) outcomes**	
Median *recurrence-free* time to death or graft failure [IQR]	6.7 [5.4, 8.6]
- graft failure, N (%)	6 (3.7%)
- death, N (%)	6 (3.7%)

*Abbreviations*:*TX* transplantation, *IQR* interquartile range, *IgAN* IgA nephropathy

**Fig. 2:**
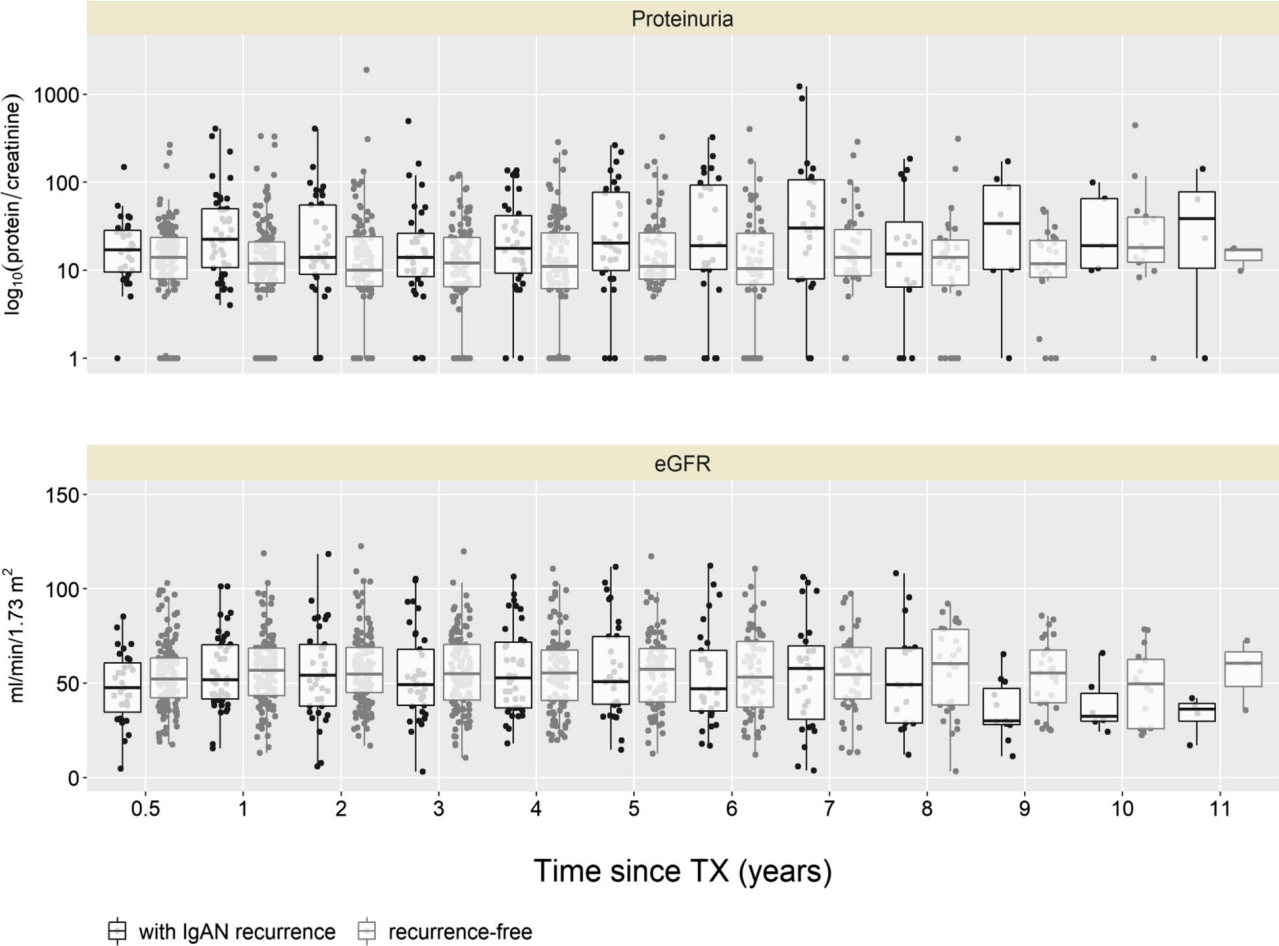
Information on proteinuria and estimated GFR over follow-up time in 161 renal transplant recipients with IgA nephropathy as underlying disease leading to TX. Abbreviations: TX – transplantation; eGFR – estimated glomerular filtration rate. Proteinuria is presented as ratio of protein to creatinine on the log scale, numerical results are given on original scale.

The proteinuria in patients with IgAN recurrence was higher at every follow-up visit than in patients without IgAN recurrence (Fig. 2). The transplant kidney function was similar in patients with and without recurrence until 7 years’ follow-up, however, after 8 years the kidney function became worse in patients with recurrence (eGFR median (interquartile range) at 8 years: 49 ml/min/1.73m^2^ (29–68) in patients with recurrence vs. 60 ml/min/1.73m^2^ (38–78) in patients without recurrence) (Fig. 2, Table 3).

**Table 3:** Clinical characteristics at recurrence, 12 months and last follow-up

At time of recurrence	R group				NR group
	All	Surveillance Bx.	Diagnostic Bx.	Clinical diagnosis	
Number of patients	43	24	13	6	–
on prednisone, N (%)	39 (90.7%)	23 (95.8%)	11 (84.6%)	5 (83.3%)	–
on RAAS blockade, N (%)	27 (62.8%)	16 (66.7%)	7 (53.8%)	4 (66.7%)	–
Median eGFR (ml/min/1.73m^2^) [IQR]	53.4 [43.4, 64]	55.7 [43.4, 63.5]	44.1 [32.6, 53.8]	61.1 [48.4, 70.2]	–
Median proteinuria^a^ (mg/mmol) [IQR]	26.5 [13.8, 49]	19.5 [12.2, 35]	68.5 [30, 113]	14.5 [12.5, 36.8]	–
Patients with glomerular hematuria	24 (55.8%)	11 (45.8%)	7 (53.8%)	6 (100%)	
At 12 months post-transplant follow-up visit					
Number of patients^b^	29	21	6	2	130
on prednisone, N (%)	26 (89.7%)	19 (90.5%)	6 (100%)	1 (50%)	102 (78.5%)
on RAAS blockade, N (%)	16 (55.2%)	12 (57.1%)	2 (33.3%)	2 (100%)	75 (57.7%)
Median eGFR (ml/min/1.73m^2^) [IQR]	49.8 [38.5, 70.5]	49.8 [40.2, 69.7]	42.2 [22.9, 50.8]	82.4 [80, 84.9]	56.8 [43.5, 68.5]
Median proteinuria^a^ (mg/mmol) [IQR]	18 [8, 37.6]	13 [7, 24.6]	37.9 [23.5, 177.7]	34.1 [19.6, 48.5]	11 [6.2, 19.9]
Patients with glomerular hematuria	9 (31%)	6 (28.6%)	2 (33.3%)	1 (50%)	0
At last follow-up visit^c^					
Number of patients	43	24	13	6	118
on prednisone, N (%)	34 (79.1%)	17 (70.8%)	11 (84.6%)	6 (100%)	76 (64.4%)
on RAAS blockade, N (%)	31 (72.1%)	21 (87.5%)	6 (46.2%)	4 (66.7%)	76 (64.4%)
Median time to last follow-up visit (years) [IQR]	7 [5, 8.8]	7 [5.6, 8.3]	6.6 [2.7, 8.9]	7.1 [7, 7.7]	6 [4.3, 8]
Median eGFR (ml/min/1.73m^2^) [IQR]	46 [24.7, 65.3]	59.5 [38.4, 65.8]	19.6 [7.8, 46]	54.9 [43.4, 65.9]	52.6 [38.3, 68.9]
Median proteinuria^a^ (mg/mmol) [IQR]	27.9 [9.2, 108.5]	11.9 [9.2, 29.1]	69.7 [15.3, 140]	47.6 [18.9, 101.2]	12.5 [6.7, 28.5]
Patients with glomerular hematuria	22 (51.2%)	9 (37.5%)	8 (61.5%)	5 (83.3%)	0

*Abbreviations*:*TX* transplantation, *IQR* interquartile range, *IgAN* IgA nephropathy, *R* recurrence, *NR* non-recurrence, *RAAS* renin-angiotensin-aldosterone system, *eGFR* estimated glomerular filtration rate, *Bx* biopsy

^a^Proteinuria is defined as protein to creatinine ratio

^b^There are two patients without 12 months post-transplant follow-up due to death or graft failure

^c^There are five patients with last follow-up visit within first 12 months post-transplant

Upon diagnosis of the recurrence, prednisone was increased or reintroduced in 15 patients and RAAS blockade was commenced in 5 patients. In 23 patients, no additional therapeutic measure was introduced. At the last follow-up visit, 79.1% of patients with recurrence and 64.4% of patients without recurrence were on prednisone, and 72.1 and 64.4% on RAAS blockade, respectively (Table 3).

### Risk factors for IgA nephropathy recurrence

**Fig. 3:**
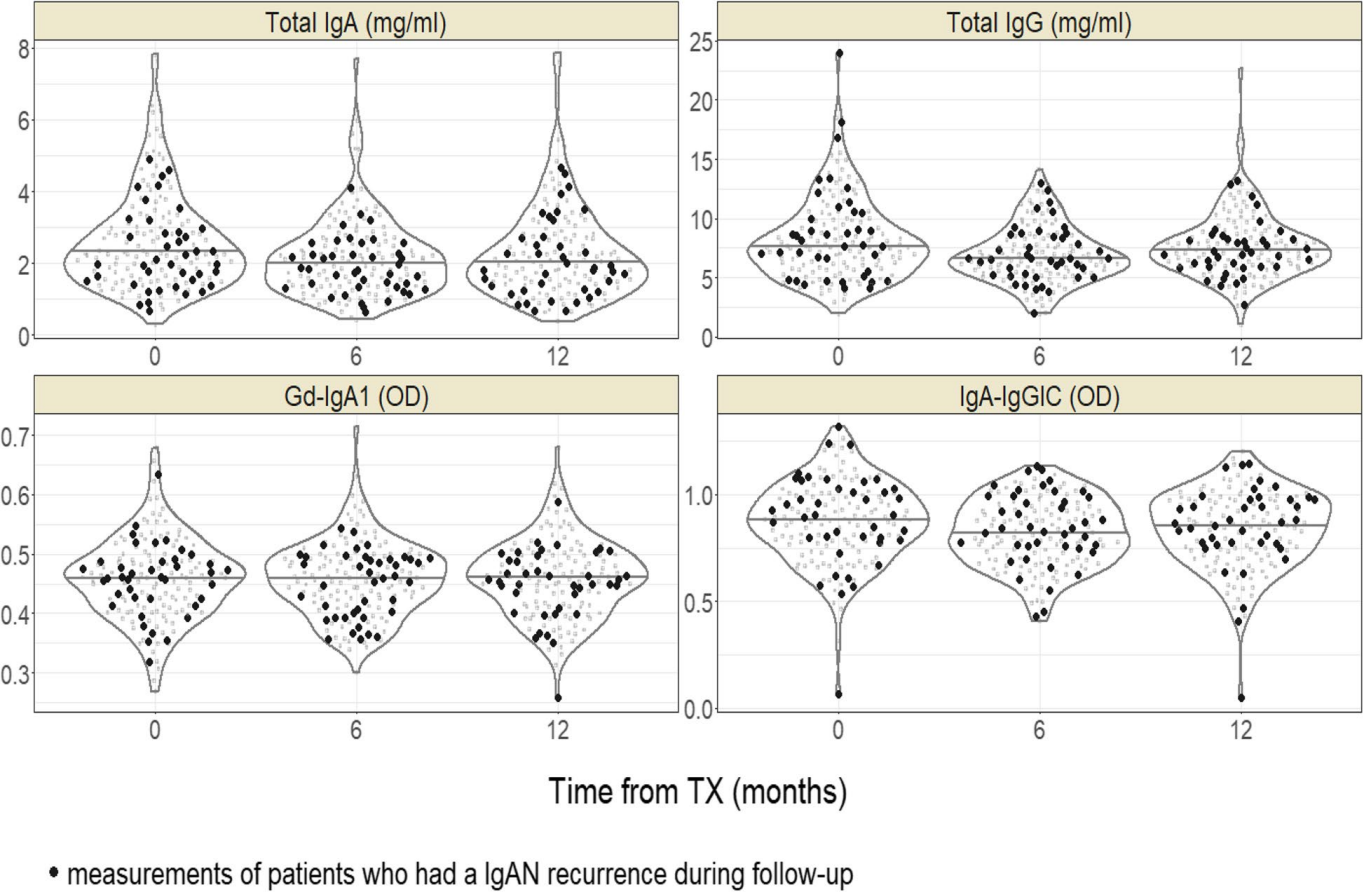
Distribution of biomarker concentrations in renal transplant recipients with IgA nephropathy as underlying disease leading to TX. Abbreviations: TX – transplantation; IgAN – IgA nephropathy; OD – optical density

Serum concentrations of total IgA, total IgG, and IgA-IgG IC decreased from baseline (just before transplantation) to 6 months, then increased until 12 months follow-up, whereas Gd-IgA1 showed very small variation during the same period (Fig. 3, Table 4). The concentration of Gd-IgA1 was similar in both recurrent and non-recurrent patient groups during the 12 months, whereas IgA-IgG IC was slightly higher in recurrent patient group.

**Table 4:** Biomarker concentration in renal transplant recipients with IgA nephropathy as underlying disease leading to TX

	Biomarker concentration, median [IQR]					
	Baseline (*n* = 159)		6 months (*n* = 158)		12 months (*n* = 155)	
	R group (*n* = 42)	NR group (*n* = 117)	R group (*n* = 42)	NR group (*n* = 116)	R group (*n* = 40)	NR group (*n* = 115)
**Total IgA (mg/ml)**	1.97 [1.53, 2.87]	2.31 [1.75, 3.33]	1.82 [1.36, 2.26]	2.13 [1.39, 2.8]	1.86 [1.26, 2.7]	2.1 [1.5, 2.86]
**Total IgG (mg/ml)**	7.96 [5.46, 10.48]	7.48 [5.88, 9.85]	6.66 [5.3, 8.6]	6.6 [5.4, 8.29]	7.04 [5.89, 8.28]	7.43 [6.06, 9.11]
**Gd-IgA1 (OD)**	0.46 [0.42, 0.49]	0.46 [0.41, 0.5]	0.46 [0.4, 0.49]	0.46 [0.43, 0.5]	0.46 [0.43, 0.49]	0.47 [0.41, 0.5]
**IgA – IgG IC (OD)**	0.91 [0.8, 1.03]	0.88 [0.76, 0.99]	0.84 [0.76, 0.99]	0.8 [0.69, 0.92]	0.87 [0.77, 0.98]	0.85 [0.71, 0.97]

*Abbreviations*:*TX* transplantation, *IgAN* IgA nephropathy, *IQR* interquartile range, *R* recurrence, *NR* non-recurrence, *OD* optical density

For the cause-specific Cox proportional hazard (PH) models, time-updated biomarker values were used. In case of Gd-IgA1, only baseline values were considered due to their low variation over the follow-up period. None of the models demonstrated a significant effect of any of the biomarkers on IgAN recurrence (Table 5). In a univariable analysis, both Gd-IgA1 (hazard ratio [HR], 0.46; 95% CI, 0.006 to 37.2; *p* = 0.73) and IgA-IgG IC (HR, 1.39; 95% CI, 0.24 to 8.03; *p* = 0.72) showed no significant influence on the risk for IgAN recurrence. Due to the low biomarker variability, time-fixed baseline biomarker models were similar to models using time-updated biomarker variables. The presence of a few positive outliers in Gd-IgA1 values at 6-month post-transplant has prevented a reasonable time-updated modelling. Any prediction of IgAN recurrence using serum concentrations of total IgA, total IgG, Gd-IgA1 and IgA-IgG IC and their longitudinal changes was not possible.

**Table 5:** Effect estimates from univariate and multivariable Cox proportional models for IgAN recurrence

HR (95%-CI) [***p***-value]	Univariate	Multivariable			
*Time-updated biomarker*					
**Total IgA (mg/ml)**	0.79 (0.59, 1.05) [0.11]	0.82 (0.62, 1.09) [0.18]	–	–	–
**Total IgG (mg/ml)**	1.003 (0.91, 1.11) [0.96]	–	1.004 (0.90, 1.12) [0.94]	–	–
**Gd-IgA1 (OD)**^**a**^	0.46 (0.006, 37.2) [0.73]	–	–	0.13 (0.001, 16.0) [0.41]	–
**IgA-IgGIC (OD)**	1.39 (0.24, 8.03) [0.72]	–	–	–	1.14 (0.20, 6.59) [0.88]
*Recipient and transplant variables*					
**Age at TX (years)**	0.96 (0.94, 0.99) [0.002]	0.96 (0.93, 0.98) [0.001]	0.96 (0.93, 0.98) [0.001]	0.95 (0.93, 0.98) [0.001]	0.96 (0.93, 0.98) [0.001]
**Female vs. Male**					
**- short (≤ one-year)**	2.83 (1.27, 6.35) [0.01]	2.49 (1.11, 5.61) [0.03]	2.52 (1.12, 5.67) [0.03]	2.67 (1.18, 6.07) [0.02]	2.52 (1.12, 5.66) [0.03]
**- long (> one-year)**	0.37 (0.05, 2.82) [0.34]	0.31 (0.04, 2.34) [0.25]	0.31 (0.04, 2.38) [0.26]	0.33 (0.04, 2.48) [0.28]	0.31 (0.04, 2.37) [0.26]
**ATG/Thymoglobulin at TX**	1.36 (0.63, 2.93) [0.44]	2.02 (0.88, 4.64) [0.10]	2.09 (0.90, 4.83) [0.09]	1.97 (0.85, 4.60) [0.12]	2.09 (0.90, 4.83) [0.09]
**Deceased vs. living**	0.94 (0.51, 1.74) [0.85]	1.28 (0.65, 2.52) [0.48]	1.25 (0.63, 2.47) [0.53]	1.22 (0.61, 2.42) [0.57]	1.24 (0.62, 2.47) [0.55]
**HLA match vs. mismatch**	0.76 (0.24, 2.46) [0.65]	0.96 (0.28, 3.27) [0.95]	0.93 (0.27, 3.20) [0.91]	0.96 (0.28, 3.27) [0.95]	0.93 (0.27, 3.17) [0.91]
**Prior kidney TX**	1.23 (0.55, 2.78) [0.61]	–	–	–	–
**RAAS exposure**	1.63 (0.87, 3.08) [0.13]	–	–	–	–
**Prednisone intake**	1.13 (0.50, 2.56) [0.76]	–	–	–	–

*Abbreviations*:*HR* hazard ratio, *CI* confidence interval, *IgAN* IgA nephropathy, *ATG* anti-thymocyte globulin, *HLA* human leukocyte antigen, *RAAS* renin-angiotensin-aldosterone system

^a^For Gd-IgA1 (OD) baseline values only are included in the multivariable model

Among the other known potential predictors of IgAN recurrence, increasing recipient age significantly reduced the risk for IgAN recurrence in multivariable analysis (HR 0.96; 95% CI, 0.93 to 0.98; *p* = 0.001). Female patients had more than twice the risk of IgAN recurrence within the first year after transplantation compared to male patients (HR 2.49; 95% CI, 1.11 to 5.61; *p* = 0.03); however, this effect was reversed, although not significantly, when looking at the period of more than 1 year after transplantation (HR 0.31; 95% CI, 0.04 to 2.34; *p* = 0.25). Induction treatment with ATG/Thymoglobulin doubled the risk of IgAN recurrence, but the effect was not significant (HR 2.02; 95% CI, 0.88 to 4.64; *p* = 0.1). Donor type, HLA full match and prior history of kidney transplantation did not influence the risk of IgAN recurrence significantly (Table 5).

In a sensitivity analysis including biopsy-proven IgAN recurrence only, results regarding biomarkers and clinical parameters were confirmed (Supplementary data, Fig. S2, Table S1).

## Discussion

In this multicentre, nationwide cohort study of kidney transplant recipients with ESKD due to IgA nephropathy, the cumulative incidence of recurrent IgAN during a median follow-up of 7 years was 26.7%. The risk for IgAN recurrence after 11 years was 27.7%. The proteinuria was higher in patients with recurrence than in patients without recurrence during the whole follow-up period despite the higher proportion of patients on RAAS blockade and prednisone at the last follow-up visit in patients with IgAN recurrence. The kidney function in patients with recurrence became worse than in patients without recurrence later in the follow-up period. The rate of graft failure in patients with recurrence was 2.8-fold higher than in patients without recurrence. In a univariable and multivariable analysis, serum concentration of total IgA, total IgG, Gd-IgA1 and IgA-IgG IC did not predict the recurrence of IgA nephropathy. Among the other known predictive factors, increasing age of recipient was protective against IgAN recurrence, whereas induction treatment with ATG/Thymoglobulin or post-transplant treatment with RAAS or prednisone did not protect against recurrence. Male gender, living donor, or higher number of HLA match did not increase the risk of IgAN recurrence.

The previously reported IgAN recurrence after transplantation is around 30%, but with a range of 9–53% among the different series depending on the biopsy policy and follow-up [17]. In our study, both clinically suspected recurrences and those diagnosed by surveillance biopsies were also included in the analysis, resulting in a slightly higher recurrence rate in a relatively short follow-up period compared to those published in previous studies [3, 5, 18].

The impact of recurrent IgAN on the graft survival has been variable in the previous reports. Some previous reports showed that IgAN recurrence has in short term little or no impact on the graft outcome during the first 10 years posttransplant [5, 19, 20], whereas others demonstrated a worse graft survival of up to 10 years. In one study the 10-year graft survival was 61% in recurrent IgAN group vs. 85.1% in non-recurrent group [21]. A recent international study reported also lower 10-year graft survival of 75% in patients with than 89% in patients without recurrence [3]. The patient survival on the other hand does not seem to be relevantly affected by recurrent IgAN. In our cohort, both the rate of graft loss and death in patients with recurrent IgAN were higher than in patients without recurrence, and the transplant kidney function also started to worsen after 7 years’ follow-up. These results suggest that the recurrent IgAN has a relevant impact on the graft function and outcome. The incidence of graft loss in our cohort was however lower than in the comparable previous studies, which might be explained by the prevalent use of prednisone and RAAS blockade. Since early steroid withdrawal has been associated with an increased risk of recurrence or graft loss because of recurrent IgAN [7, 22], many patients in our cohort seem to remain on the permanent therapy with prednisone, i.e. triple immunosuppressive therapy. The RAAS blockade was also widely used, even in patients without recurrence, since RAAS blockade is so far one of the most important supportive therapies in patients with IgAN [23].

Previous studies have shown the predictive ability of biomarkers, such as serum Gd-IgA1 or autoantibodies specific for Gd-IgA1, for the recurrence of IgAN [12, 14]. In the study of Berthelot et al. [12], higher concentrations of both Gd-IgA1 and IgA-IgG IC at the time of transplantation were predictive for recurrence. In the study of Berthoux et al. [14], only serum Gd-IgA1 specific IgG autoantibodies at the time of transplantation was predictive for IgAN recurrence, and the level of Gd-IgA1 did not associate with any outcome. In our study, both pre- and posttransplant measurements of Gd-IgA1, IgA-IgG IC and total IgA and IgG could not demonstrate any significant effect on IgAN recurrence, although the number of patients was higher than in previous studies. We do not have clear explanations for the discrepant results among these 3 studies including ours. The ELISA-based measurement methods were all similar in 3 studies. Previous studies have enrolled patients from earlier periods of 2000–2012 [12] and 1985–2007 [14], and this might have led to discrepant biomarker responses due to differences in patient management including immunosuppressive regimen. Higher proportion of Caucasian in our study (91.9%) compared with previous study of Berthelot et al. (57%) may also explain the different results. The results of 3 studies showed that there are so far no reliable clinical parameters or biomarkers to predict the recurrence of IgAN after transplantation.

The biomarker concentration evaluated in our study showed low variability over the first year posttransplant (pre-transplant, 6 months & 12 months), which is consistent to our previous study [16]. We have previously shown that the concentration of Gd-IgA1 and IgA-IgG IC decreased during the first 3 months posttransplant under high dose of prednisone and restored to pretransplant level at 6 months under gradual reduction of prednisone. Most of our patients were on a stable low dose of prednisone at 6 months posttransplant and remained on a triple immunosuppression including low dose prednisone thereafter, which may have led to the low variability in serum concentrations of biomarkers.

Our study has several strengths. First, all the clinical data and biological samples were collected prospectively in a nationwide transplant cohort study with detailed follow-up data. The number of patients was also higher than that of most previous studies. Second, since our cohort study started in 2008, the patients represent the patient population with current standard immunosuppressive regimen and practice, while most previous studies included patients transplanted in the 1980s and 1990s. This may explain at least partly the discrepant results of our study compared with previous studies. Third, we have measured the biomarkers at 3 different time-points, which provided additional information on the posttransplant course of biomarkers.

Our study has also several limitations. First, the follow-up period was relatively short to adequately evaluate the impact of IgAN recurrence on the graft and patient outcome. Second, not all recurrences were diagnosed by transplant kidney biopsy. This was due to the prospective real-life setting of our study, where different local biopsy policies applied. We however performed the adjudicated final diagnosis procedure, in which all the posttransplant data were meticulously reviewed to define the IgAN recurrence. Third, we cannot exclude pre-existing IgA deposits in the donor kidneys, in case there were only IgA deposits without further light microscopic changes in the transplant kidneys, since the zero-hour biopsy before transplantation has not been performed systematically in our cohort. Fourth, we did not measure the biomarker concentrations at the time of the IgAN recurrence. In addition, we measured IgG against IgA, but not specifically against Gd-IgA1.

In conclusion, our cohort study including patients on current standard immunosuppressive regimen and current standard practice showed that the recurrence of IgAN after transplantation has a relevant impact on the graft function and outcome. The rate of graft loss was however, compared with the previous studies, relatively low. In our study population, we were not able to confirm the predictive value of biomarkers from the previous studies for the recurrence of IgAN.

## Supplementary Information

**Additional file 1.**

**Additional file 2.**

**Additional file 3.**

**Additional file 4.**

**Additional file 5.**

## Data Availability

All data generated or analysed during this study are included in this published article and its supplementary information files.
