# Supplementary material for: Recurrence of IgA nephropathy after kidney transplantation: experience from the Swiss transplant cohort study
Source: BMC Nephrol. 2022 May 10;23:178. doi: 10.1186/s12882-022-02802-x (PMC9088042; doi:10.1186/s12882-022-02802-x)
Supplement: Supplementary file 1 — Additional file 1. [file 12882_2022_2802_MOESM1_ESM.docx]

# **Supplementary data**

**Figure S1:** Probability of IgAN recurrence, death and graft failure post-transplant in the study population.


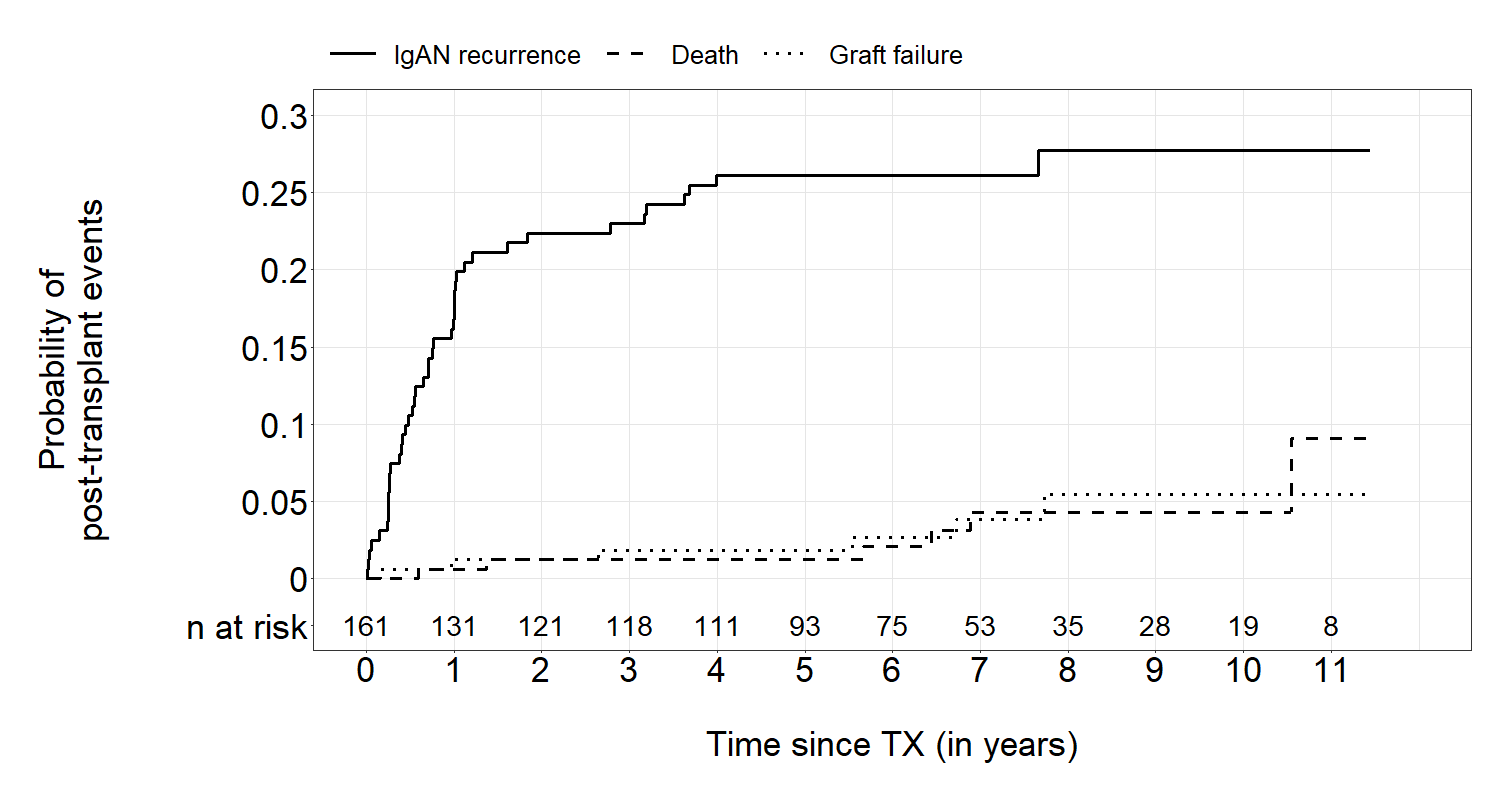


Abbreviations: TX – transplantation; IgAN – IgA nephropathy.

**Figure S2:** Probability of biopsy-proven IgAN recurrence, death and graft failure post-transplant in the study population.


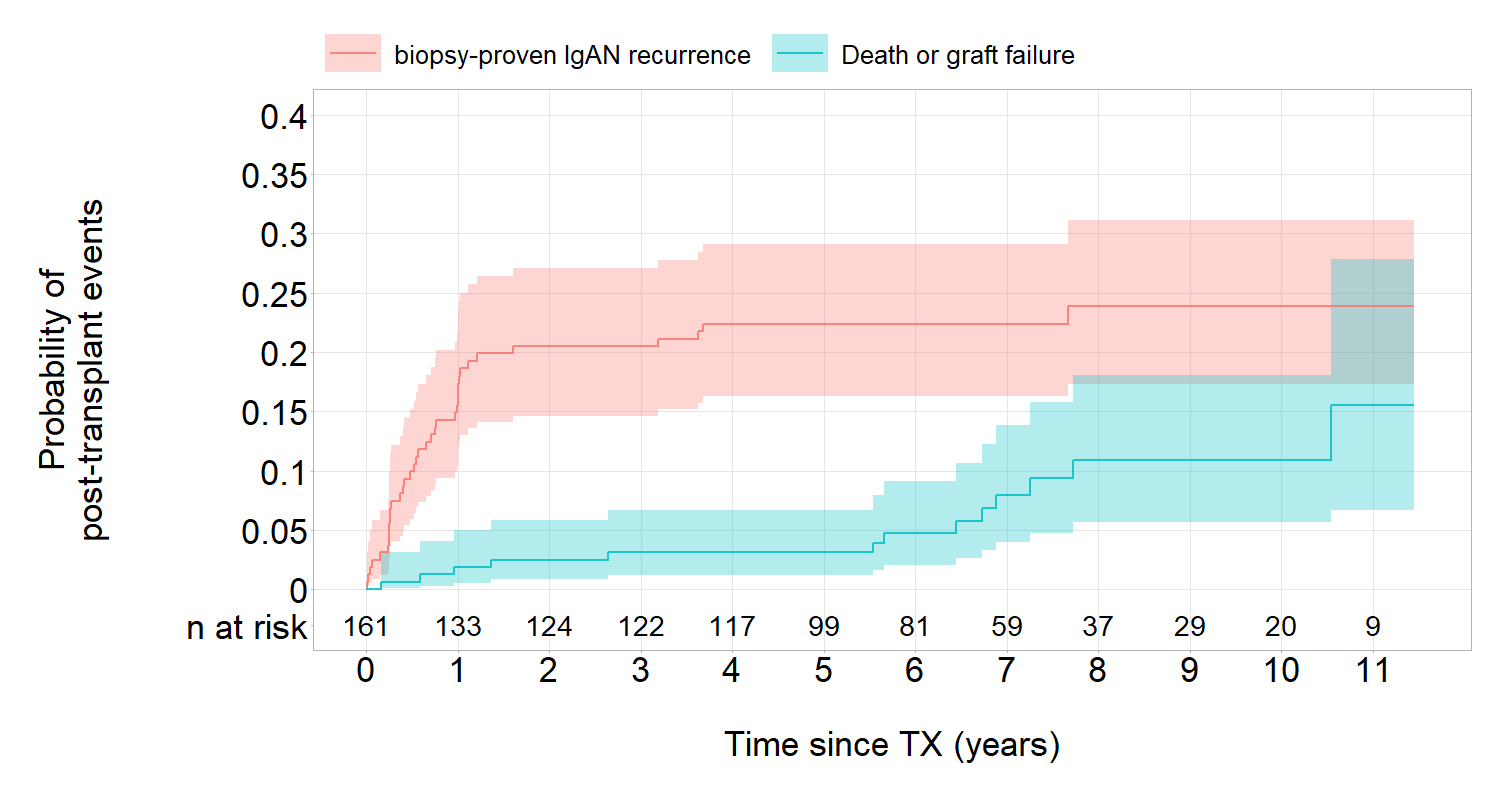


Abbreviations: TX – transplantation; IgAN – IgA nephropathy.

**Table S1:** Effect estimates from a cause-specific Cox proportional hazard models for biopsy-proven IgAN recurrence.

| **HR**  **(95%-CI)**  **[p-value]** | **Univariate** | **Multivariable** | | | |
| --- | --- | --- | --- | --- | --- |
| *Time-updated biomarker* | | | | | |
| **Total IgA (mg/m)** | 0.75  (0.55, 1.04) [0.08] | 0.79  (0.57, 1.07) [0.13] | - | - | - |
| **Total IgG (mg/ml)** | 1.02  (0.92, 1.13) [0.73] | - | 1.01  (0.91, 1.13) [0.80] | - | - |
| **Gd-IgA1 (OD) *** | 0.36  (0.003, 42.1) [0.67] | - | - | 0.09  (0.00, 17.0) [0.37] | - |
| **IgA-IgGIC (OD)** | 1.95  (0.28, 13.48) [0.50] | - | - | - | 1.58  (0.22, 11.10) [0.65] |
| *Recipient and transplant variables* | | | | | |
| ***Age at TX (years)*** | 0.97  (0.95, 0.99) [0.02] | 0.97  (0.94, 0.99) [0.03] | 0.97  (0.94, 0.995) [0.02] | 0.97  (0.94, 0.99) [0.02] | 0.97  (0.94, 0.99) [0.02] |
| **Female vs. Male**  **- short (≤ one-year)**  **- long (> one-year)** | 3.21  (1.41, 7.32)  [0.01]  0.53  (0.07, 4.09)  [0.54] | 2.92  (1.27, 6.70) [0.01]  0.47  (0.06, 3.68) [0.47] | 2.92  (1.27, 6.70) [0.01]  0.47  (0.06, 3.68) [0.47] | 3.13  (1.35, 7.25) [0.01]  0.50  (0.06, 3.91) [0.51] | 2.90  (1.26, 6.65) [0.01]  0.48  (0.06, 3.70) [0.48] |
| **ATG/Thymoglobulin at TX** | 1.38  (0.61, 3.16)  [0.44] | 1.96  (0.80, 4.78) [0.14] | 2.03  (0.82, 5.00) [0.13] | 1.90  (0.77, 4.72) [0.16] | 2.03  (0.82, 5.00) [0.13] |
| **Deceased vs. living** | 0.88  (0.45, 1.71)  [0.70] | 1.08  (0.52, 2.27) [0.83] | 1.05  (0.50, 2.21) [0.90] | 1.02  (0.48, 2.15) [0.96] | 1.02  (0.48, 2.16) [0.97] |
| **HLA match vs. mismatch** | 0.92  (0.28, 2.98)  [0.88] | 1.08  (0.31, 3.74) [0.90] | 1.00  (0.28, 3.50) [1.00] | 1.04  (0.30, 3.60) [0.95] | 0.99  (0.29, 3.44) [0.99] |
| **RAAS exposure** | 1.49  (0.74, 2.99)  [0.27] | - | - | - | - |
| **Prednisone intake** | 0.97  (0.40, 2.32) [0.94] | - | - | - | - |

Abbreviations: HR – hazard ratio; CI – confidence interval; IgAN – IgA nephropathy; ATG – anti-thymocyte globulin; HLA – human leukocyte antigen; RAAS - renin-angiotensin-aldosterone system.

* For Gd-IgA1 (OD) baseline values only are included in the multivariable model.
